# Performance of real-time polymerase chain reaction and Kato-Katz for diagnosing soil-transmitted helminth infections and evaluating treatment efficacy of emodepside in randomized controlled trials

**DOI:** 10.1371/journal.pntd.0012872

**Published:** 2025-02-18

**Authors:** Christian N. Lotz, Emmanuel C. Mrimi, Pierre H. H. Schneeberger, Said M. Ali, Jan Hattendorf, Jennifer Keiser

**Affiliations:** 1 Swiss Tropical and Public Health Institute, Allschwil, Switzerland; 2 University of Basel, Basel, Switzerland; 3 Ifakara Health Institute, Morogoro, Tanzania; 4 Public Health Laboratory-Ivo de Carneri, Chake Chake, Pemba, Tanzania; Consejo Nacional de Investigaciones Cientificas y Tecnicas, Fundación Mundo Sano, ARGENTINA

## Abstract

**Background:**

The World Health Organization recommends the use of the microscopy-based Kato-Katz thick smear for diagnosing soil-transmitted helminth (STH) infections. Despite its simplicity and cost-effectiveness, the Kato-Katz method faces challenges, including reader subjectivity and reduced sensitivity. Real-time polymerase chain reaction (qPCR) technology offers standardized readouts and higher sensitivity, making it suitable for STH diagnosis and monitoring the treatment efficacy of emodepside within the framework of randomized controlled trials.

**Methodology/Principal findings:**

We evaluated the performance of Kato-Katz versus qPCR for assessing treatment efficacy in terms of cure rates, of single doses of 5, 10, 15, 20, 25 and 30 mg of emodepside compared to 400 mg albendazole. Spearman’s rank correlation coefficient examined the correlation between STH eggs per gram in stool samples and qPCR Ct values. Diagnostic sensitivity of qPCR was calculated using a Bayesian latent class modelling approach with data from *Ascaris lumbricoides* infections. Agreement between Kato-Katz and qPCR at baseline was 93.57% for *Trichuris trichiura*, and 73.49% for both hookworm and *A. lumbricoides*. For the latter helminth qPCR demonstrated higher sensitivity (85.00% *vs*. 47.70%) and slightly lower specificity (93.40% *vs*. 99.40%) compared to Kato-Katz. We observed a fair to moderate agreement with negative correlation between Ct values and Kato-Katz egg counts. Treatment efficacy, as assessed by qPCR, was lower for all doses of emodepside and albendazole compared to Kato-Katz. Nonetheless, emodepside demonstrated higher cure rates against *T. trichiura* and *A. lumbricoides* infections compared to albendazole.

**Conclusion/ Significance:**

Our study confirmed that qPCR is a sensitive diagnostic method for diagnosing STH infections compared to Kato-Katz and serves as a valuable tool for determining treatment efficacy in clinical trials. Furthermore, qPCR confirmed the better treatment efficacy of emodepside compared to albendazole, despite indicating lower cure rates than Kato-Katz.

## Author summary

Soil-transmitted helminthiasis, a widespread neglected tropical disease, affects over 1.5 billion people globally. This disease is caused by *Trichuris trichiura*, hookworms, and *Ascaris lumbricoides*. Moderate to heavy infections can lead to symptoms such as abdominal pain, diarrhea, and dysentery, and if untreated, can result in serious complications like anemia, chronic malnutrition, and developmental delays. Our research compared the diagnostic performance of the traditional microscope based Kato-Katz technique *versus* real-time polymerase chain reaction (qPCR) for detecting soil-transmitted helminths (STHs). We tested these diagnostic methods in a framework of randomized controlled trials assessing the efficacy of a new treatment, emodepside, against *T. trichiura* and hookworm infections. qPCR proved to be more sensitive than Kato-Katz in detecting STH infections, especially for *A. lumbricoides*. Although qPCR showed lower cure rates for both treatments compared to Kato-Katz, it confirmed that emodepside was highly efficacious against all STHs. In summary, our study confirmed that qPCR is a sensitive diagnostic tool for STH infections, ensuring a more effective disease management.

## Introduction

Soil-transmitted helminthiasis is the most prevalent neglected tropical disease, affecting over 1.5 billion people globally [1]. Soil-transmitted helminth (STH) infections are caused mainly by *Trichuris trichiura*, hookworms (*Necator americanus* and *Ancylostoma duodenale*) and *Ascaris lumbricoides*. Moderate-to-heavy intensity infection with STHs can be the cause of symptoms such as abdominal pain, diarrhea and dysentery [2,3]. If left untreated, they may lead to further chronic complications such as anemia, chronic malnutrition, physical and cognitive retardation [2,3].

Recently, the World Health Organization has set its agenda to eliminate STH as a public health problem for population at risk, using periodic large-scale administrations of single dose benzimidazoles (albendazole and mebendazole), by 2030 [4]. To achieve this goal a simple, cost-effective, and sensitive diagnostic technique is needed to monitor progress and determine the justification for scaling down or discontinuing these mass drug administration programs [4]. For nearly half a century now, this technique is Kato-Katz, which involves analyzing thick smears of stool under a microscope [5,6].

Despite its advantages, being simple, inexpensive and suitable for detecting STHs moderate-to-heavy intensity of infections, Kato-Katz thick smear has various shortcomings. Its evaluation is subjective and highly dependent on the reader’s experience and skills, especially given the declining expertise in bright-field microscopy [7]. Additionally, there are sensitivity issues as eggs are unevenly distributed within a single stool sample, and day-to-day fluctuations in egg excretions are common [8]. These issues paired with the small amount of stool analyzed, lead to missing low-intensity infections, resulting in an underestimation of prevalence and an overestimation of cure rates in studies where only infected individuals are eligible [6,9,10].

These limitations can be overcome by using real-time polymerase chain reaction (qPCR), a molecular diagnostic method that has gained prominence in recent decades and was already frequently used in STH research, including clinical trials [10]. qPCR offers an objective readout through fluorescence signals, with their intensity directly correlated to the amount of target DNA. Furthermore, it can detect multiple species simultaneously while achieving higher specificity and sensitivity compared to Kato-Katz. qPCR can also distinguish between morphologically identical species, such as the eggs of *T. trichiura* and *Trichuris suis* [11]. While it is optimal to apply qPCR on fresh stool samples, they can also be stored in ethanol or frozen for later use. Since it is DNA-based, long-term storage of samples is possible [12–14].

Emodepside is a novel key player in the anthelminthic drug armamentarium. In 2021, two phase 2a clinical dose-finding studies conducted on Pemba Island, Tanzania, investigated the efficacy of emodepside in treating hookworm and *T. trichiura* infections. The studies, analyzed via Kato-Katz demonstrated that emodepside had high cure rates for *T. trichiura*, ranging from 83-100%, compared to 17% with albendazole. Additionally, a dose-dependent relationship was observed for hookworm, with cure rates of 32% at 5 mg and 95% at 30 mg, significantly outperforming the placebo- (14%) and albendazole (70%) groups [15].

The aim of this study is to provide further evidence on the performance of qPCR compared to the Kato-Katz method for detecting STH infections. Additionally, using an existing clinical trial framework, we aim to evaluate the previously observed high treatment efficacy of emodepside against all STHs infection using qPCR as a second diagnostic tool.

## Methods

### Ethics statement

This analysis utilized data and samples collected during the clinical trials, registered on clinicaltrials.gov under reference NCT05017194. Approval for ethical considerations was granted by the Zanzibar Ministry of Health (Ref: NO.ZAHREC/03/JUNE/2021/11), the Zanzibar Food and Drug Agency (1.0 V1.0; 08.10.2020), and the Ethics Committee Northwest and Central Switzerland (AO_2021–00028). The trials adhered to the principles outlined in the Declaration of Helsinki and followed the guidelines of Good Clinical Practice. Written informed consent was obtained from all participants prior to study start.

### Study design and setting

The two phase 2a, single-blind, dose-ranging, randomized, placebo-controlled trials were conducted in five administrative areas (Mapofu, Mtemani, Piki, Njuguni, and Ndagoni) on Pemba Island, Tanzania, from August 2, 2021, to December 10, 2021 [15]. Briefly, a total of 442 adults aged between 18 and 45 years, with an infection intensity of at least 48 eggs per gram of stool (EPG), were included in the studies. Depending on their infection status, eligible participants were assigned to either the *T. trichiura* or hookworm trial. For treatment, participants were randomly assigned to receive a single dose of 5, 10, 15, 20, 25 or 30 mg emodepside; 400 mg of albendazole; or placebo.

### Sample collection and laboratory procedures

People who provided informed consent were invited to submit two fresh stool samples pre-treatment (baseline 1 and baseline 2) and another two fresh stool samples 14-21 days post-treatment (follow-up 1 and follow-up 2). For analyses, stool samples were cold-chained and transported to the nearby laboratory at the Public Health Laboratory - Ivo de Carneri. Identification of STH eggs in the stool samples was done using duplicate Kato-Katz thick smears [5]. These smears were examined by experienced technicians under a light microscope within a maximum of 60 minutes after preparation. Eggs were counted and recorded for each STH species separately. The mean of the two readings was multiplied by a factor of 24 to obtain a measure of intensity, expressed by EPG of stool [16].

The remaining stool samples after Kato-Katz analysis, were preserved for qPCR analysis. Approximately, 500 µl of the stool sample was stored in -20°C and 500 µl in 2 ml 70% ethanol, prior to shipping to the Swiss Tropical and Public Health Institute (Swiss TPH) in Allschwil, Switzerland.

For DNA extraction, stool stored in ethanol was homogenized and centrifuged at 15000 x g for 5 min to obtain approximately 150 mg of starting material, mirroring the process for frozen samples. DNA from both ethanol-preserved and frozen samples was extracted using DNeasy PowerSoil Pro Kits (Qiagen; Hilden, Germany), and eluted in 60 µl. The DNA was measured using a NanoDrop One/OneC (ThermoFisher, Switzerland), ensuring successful extraction. Successful extractions were defined as those with a DNA concentration exceeding 25 ng/µL and a 260/230 nm absorbance ratio of 2 ± 0.2. If this criterion was not met, the extraction was repeated. For detecting helminthic DNA a multiplexed qPCR was used, based on the method of Keller et al. [10]. Primers and probes were procured from Microsynth, Switzerland, the TaqMan GeneExpression MasterMix from ThermoFisher, Switzerland. The primers and probes were designed to detect the 18S rRNA gene of *T. trichiura*, the internal transcribed spacer of *A. lumbricoides,* and the internal transcribed spacer of *N. americanus* (S1 Table). Since the prevalence of *Ancylostoma duodenale* on Pemba Island is considered low, no qPCR detection was performed on this species [17].

For the qPCR reaction, 5 μL of TaqMan Gene Expression MasterMix (ThermoFisher, Switzerland) was mixed with the primers and probes (S1 Table). DNAse-free water (Gibco, Switzerland) was added to reach a volume of 8 μL, followed by 2 μL of the sample or controls. The mixture was thoroughly combined and transferred to a 384 well plate (ThermoScientific, Switzerland). Amplification was carried out on the CFX Opus 384 with the following program: initial pre-amplification at 50 °C for 2 minutes, then 95 °C for 10 minutes, followed by 50 cycles of 15 seconds at 95 °C and 1 minute at 58 °C. Each sample was run in duplicates for accuracy. Controls included ultrapure water and standards with 1000 and 1,000,000 gene copy numbers (GCN)/µL for each species.

Standard curves for the different amplicons were established using a dilution series of plasmids containing the relevant DNA sequences. DNA derived from the stool of healthy uninfected individuals was used as a negative control. Cycle threshold (Ct) values were plotted against the logarithm of starting DNA quantities. The Ct, indicating significant amplification, occurs when the signal surpasses a predetermined threshold [18,19]. The calibration curves had to result in a duplication efficiency between 80 and 110% and an R-square value > 0.99 to be considered valid (S1 Table). Each dilution series underwent testing with and without other targets to rule out cross-reactions and/or inhibitions between primers, probes or different targets. The lower limit of quantification (LLOQ) was established at the lowest point on our calibration curve, where the signal remains at least five times higher than the blank.

### Statistical analysis

#### Data Entry, cleaning, and quality assurance for Kato-Katz and qPCR analysis.

Data from the Kato-Katz thick smear readout were double entered into a database (Access 2003, Microsoft) by two staff members using CommCare (Dimagi, Cambridge, MA, USA). The Data Compare tool of Commcare was used to crosscheck both entries, and discrepancies were corrected by referring to the original data sheets.

Data cleaning and quality checking for the qPCR samples were done using the CFX Maestro software (Bio-Rad Laboratories, Inc, Hercules, CA, USA). A sample was considered positive for an STH if the curve was sigmoidal, the fluorescence signal exceeded a set threshold, and the Ct fell within the range of the calibration curve for the corresponding helminth. Following these criteria, the Ct values of the samples were transcribed through the linear equation of the calibration curves into DNA copies/µL using the cycle number when they surpassed the threshold. Patients were classified as infected for Kato-Katz or qPCR if one sample was positive at any point during Baseline 1 or 2. Only stool samples that were analyzed by both Kato-Katz and qPCR were included in this study.

#### Correlation between microscopic egg count and qPCR Ct values.

We analyzed the correlation between EPG and qPCR Ct values, as a base of sensitivity and specificity estimates. We employed Spearman’s rank correlation coefficient (r_s_) to assess potential correlation in samples identified as positive by all diagnostic methods, with further stratification by STH species and sampling time points. The degree of agreement was categorized as follows: “none” (0–0.20), “fair” (0.21–0.40), “moderate” (0.41–0.60), “substantial” (0.61–0.80), and “almost perfect” (0.81–1.00) [20,21].

#### Diagnostic performance of Kato-Katz and qPCR in terms of sensitivity and specificity.

To account for the absence of a gold standard test for STHs, characterized by imperfect sensitivity and specificity, we utilized a Bayesian latent class modelling two for two correlated tests in two populations without a gold standard approach to estimate diagnostic accuracy [22]. The model is overparameterized; therefore, we used highly informative beta priors for the specificity of the Kato-Katz method (mode = 99.5%, 95% sure >99%). Specifically, data from *A. lumbricoides* infections were used to estimate the sensitivity and specificity of qPCR compared to the Kato-Katz method, as no screening criteria regarding its infection status applied.

Prior to calculation, we assumed that qPCR and Kato-Katz are conditionally dependent since both methods detect parasites. However, qPCR detects DNA associated with both worm and egg material, while Kato-Katz detect solely the eggs, thus introducing a degree of independence from Kato-Katz. The final model was implemented using OpenBUGS software (version 3.2.3) using the R2OpenBUGS and mcmcplots packages in R.

## Results

### Overall positivity agreement according to Kato‑Katz and qPCR for all four examination time points

A total of 249 stool samples at baseline, and 235 samples 14–21 days post-treatment were collected and analysed using both the Kato-Katz method and qPCR. Among all stool samples, 299 were positive for *T. trichiura* according to Kato-Katz, while 385 samples were positive according to qPCR. There were 114 samples (23.55%) with discordant results: 14 samples were positive only by Kato-Katz, and 100 samples were positive only by qPCR (Table 1). For baseline there was a concordance of 93.6% while for the follow-up a concordance of 58.3% was observed. For participants positive for hookworm, 146 out of 484 samples (30.17%) exhibited discordant results. Specifically, 25 samples were positive only by Kato-Katz, and 121 samples only by qPCR. Here concordance for both baseline and follow-up was around 70% each. Regarding *A. lumbricoides*-positive participants, 100 samples were positive according to Kato-Katz, while 150 samples were positive according to qPCR. 90 out of 484 samples (18.60%) had discordant results: 20 samples were positive only by Kato-Katz, and 70 samples were positive only by qPCR. The individual concordance of baseline was 73.5% and follow-up was 89.8%. This resulted in a calculated prevalence of 69.8% (95% CI: 54.9–96.7) during baseline and 15.4% (95% CI: 8.0–25.8) during follow-up. Overall qPCR identified more than twice the number of stool samples as STH-positive compared to the Kato-Katz method.

**Table 1:** Positivity agreement according to Kato-Katz and qPCR for all four-examination time points for T. trichiura, hookworm and A. lumbricoides.

	Time point	Kato-Katz	qPCR	
**qPCR positive**	**qPCR negative**
*T. trichiura*	Baseline	Kato-Katz positive	231 (92.8%)	8 (3.2%)
		Kato-Katz negative	8 (3.2%)	2 (0.8%)
	Follow-up	Kato-Katz positive	54 (23.0%)	6 (2.6%)
		Kato-Katz negative	92 (39.1%)	83 (35.3%)
Hookworm	Baseline	Kato-Katz positive	97 (39%)	16 (6.4%)
		Kato-Katz negative	50 (20.1%)	86 (34.5%)
	Follow-up	Kato-Katz positive	36 (15.3%)	9 (3.8%)
		Kato-Katz negative	71 (30.2%)	119 (50.6%)
*A. lumbricoides*	Baseline	Kato-Katz positive	68 (27.3%)	14 (5.6%)
		Kato-Katz negative	52 (20.9%)	115 (46.2%)
	Follow-up	Kato-Katz positive	12 (5.1%)	6 (2.6%)
		Kato-Katz negative	18 (7.7%)	199 (84.7%)

### Sensitivity of qPCR using Kato-Katz as a reference method

**Fig 1:**
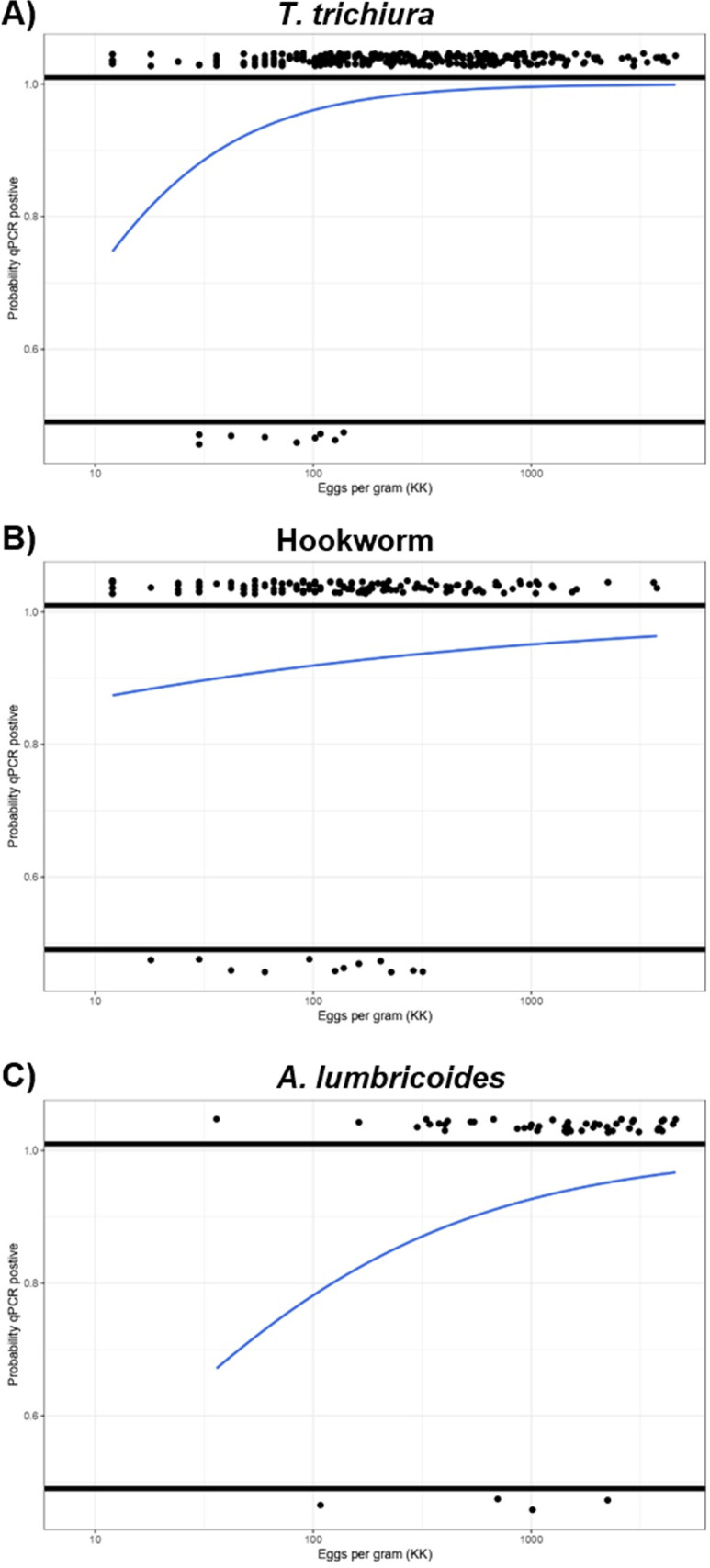
Probability of positive qPCR Ct value in relation to increasing *T. trichiura* (Panel A), hookworm (Panel B) and *A. lumbricoides* (Panel C) egg counts from Kato-Katz thick smear microscopy. Black dots: *T. trichiura* (Panel A), hookworm (Panel B) and *A. lumbricoides* (Panel C) eggs counts in qPCR-positive (top) and qPCR-negative (bottom) samples. Blue line: probability curve for GCN/ µl > 2 (Panel A), GCN/ µl > 20 (Panel B) and GCN/ µl > 20 (Panel C) to become positive if the corresponding Kato-Katz is the reference test.

The probability of detecting STH DNA increased proportionally with an increase in STH egg counts (Fig 1). In *T. trichiura*-positive samples, the relative DNA detection rates increase with infection intensity, approaching 100% at heavy intensity of infection (Fig 1A). This shape is less pronounced for *A. lumbricoides*- (Fig 1C) and hookworm-positive samples (Fig 1B).

In terms of diagnostic sensitivity, qPCR demonstrated higher sensitivity (85.0, 95% Credible Intervals (CrI): 61.5–97.9) for detecting *A. lumbricoides* compared to Kato-Katz (47.7, 95% CrI: 32.7–60.6). However, Kato-Katz exhibited slightly higher specificity (99.4, 95% CrI: 98.8–99.8, representing the informative prior distribution) for *A. lumbricoides* detection compared to qPCR (93.4, 95% CrI: 87.0-99.2).

### Correlation between microscopic egg counts and qPCR Ct values

Table 2 summarizes the correlation between positive parasite loads, assessed through microscopic egg counts, with qPCR Ct values. A significant negative correlation was observed between most qPCR Ct values and microscopic egg counts. Specifically, for *T. trichiura* positive samples, correlations ranged from fair to moderate: follow-up day 2 (*r*_*s*_ = -0.44), baseline day 1 (*r*_*s*_ = -0.29), and baseline day 2 (*r*_*s*_ = -0.21). Hookworm positive samples also showed a moderate negative correlation on follow-up day 1 (*r*_*s*_ = -0.53) and day 2 (*r*_*s*_ = -0.46). For *A. lumbricoides* positive samples, fair negative correlations were found at baseline day 1 (*r*_*s*_ = -0.39) and day 2 (*r*_*s*_ = -0.30).

**Table 2:** Spearman’s rank correlations between EPG and qPCR Ct values for each time point among positive tests for each STH species.

		No. of positive test results	*ρ*	*P*-value
***T. trichiura***	Baseline day 1	205	-0.29	<0.001
	Baseline day 2	196	-0.21	0.003
	Follow-up day 1	43	-0.16	0.31
	Follow-up day 2	42	-0.44	0.004
**Hookworm**	Baseline day 1	73	-0.09	0.44
	Baseline day 2	79	-0.17	0.13
	Follow-up day 1	24	-0.53	0.007
	Follow-up day 2	25	-0.46	0.02
***A. lumbricoides***	Baseline day 1	68	-0.39	<0.001
	Baseline day 2	67	-0.30	0.01
	Follow-up day 1	14	-0.10	0.73
	Follow-up day 2	14	-0.38	0.19

### Cure rates according to Kato-Katz and qPCR

Cure rates for treatment with placebo, different dosages of emodepside and albendazole were calculated based on the Kato-Katz or qPCR diagnosis. The overall cure rates of emodepside against all STH, as measured by qPCR, were generally lower compared to those determined by the Kato-Katz method (Table 3). For participants infected with *T. trichiura* who received emodepside, the cure rate was on average 2.2 times higher when assessed using the Kato-Katz technique compared to qPCR, ranging from 5 mg and a cure rate of 77.27% with Kato-Katz versus 50% qPCR to 94.44% versus 39.41% for the 20 mg emodepside dosage.

**Table 3:** Comparison of treatment efficacy in terms of cure rates between Placebo, emodepside treatment arms and albendazole by diagnostic approach (Kato-Katz versus qPCR).

		Placebo	5 mg	10 mg	15 mg	20 mg	25 mg	30 mg	Albendazole
***T. trichiura***									
Kato-Katz	No. cured/ No. positive baseline	2/19	17/22	19/20	16/16	17/18	17/18	17/19	3/18
	Cure rates [%] (95% CI)	10.53 (2.94–31.39)	77.27 (56.56–89.88)	95.00 (76.39–99.11)	100 (80.64–100)	94.44 (74.24–99.01)	94.44 (74.24–99.01)	89.47 (68.61–97.06)	16.67 (5.84–39.22)
qPCR	No. cured/ No. positive baseline	1/19	10/20	8/19	8/16	6/18	5/17	8/19	2/17
	Cure rates [%] (95% CI)	5.26 (0.94–24.64)	50.00 (29.93–70.07)	42.11 (23.14–63.72)	50.00 (28.00–72.00)	33.33 (16.28–56.25)	39.41 (13.28–53.13)	42.11 (23.14–63.72)	11.76 (3.29–34.34)
**Hookworm**									
Kato-Katz	No. cured/ No. positive baseline	1/12	3/12	4/9	12/16	11/16	12/13	9/9	10/12
	Cure rates [%] (95% CI)	8.33 (1.49–35.39)	25.00 (8.89–53.23)	44.44 (18.88–73.33)	75.00 (50.50–89.82)	68.75 (44.40–85.84)	92.31 (66.69–98.63)	100 (70.09–100)	83.33 (55.20–95.30)
qPCR	No. cured/ No. positive baseline	1/8	1/8	1/8	10/12	7/15	5/13	6/9	5/12
	Cure rates [%] (95% CI)	12.50 (2.24–7.09)	12.50 (2.24–7.09)	12.50 (2.24–7.09)	83.33 (55.20–95.30)	46.67 (24.81–69.88)	38.46 (17.71–64.48)	66.67 (35.42–87.94)	41.67 (19.33–68.05)
***A. lumbricoides***									
Kato-Katz	No. cured/ No. positive baseline	1/13	13/15	5/5	6/6	12/13	11/11	10/10	9/9
	Cure rates [%] (95% CI)	7.69 (1.37–33.31)	86.67 (62.12–96.26)	100 (56.55 –100)	100 (60.97–100)	92.31 (66.69–98.63)	100 (74.12–100)	100 (72.25–100)	100 (70.09–100)
qPCR	No. cured/ No. positive baseline	4/22	19/20	12/12	19/21	18/22	15/18	9/15	16/20
	Cure rates [%] (95% CI)	18.18 (7.31–38.52)	95.00 (76.39–99.11)	100 (75.75–100)	90.48 (71.09–97.35)	81.82 (61.48–92.69)	83.33 (60.78–94.16)	60.00 (35.75–80.18)	60.00 (58.40–91.93)

Despite the lower cure rates observed with qPCR, all doses of emodepside (cure rate average of 42.83%) showed significantly higher cure rates against *T. trichiura* compared to the albendazole treatment group (11.76%) and the dose-response curves for both qPCR and Kato-Katz results followed the same pattern. Similarly, for participants infected with hookworm and treated with emodepside, the cure rates were higher when diagnosed using Kato-Katz than with qPCR and both qPCR and Kato-Katz results showed positive dose-response curves. However, no significant difference in cure rates was observed between emodepside (49.52%) and albendazole (41.67%) treatments for hookworm-infected participants when using qPCR for diagnosis. For *A. lumbricoides*, the cure rates for both emodepside and albendazole were high when diagnosed using either Kato-Katz or qPCR, with rates of 96.50% and 100% for Kato-Katz, compared to 85.11% and 60% for qPCR, across most treatment arms

## Discussion

In this study, we compared the performance of Kato-Katz and qPCR methods for STH diagnosis, examining both qualitative and quantitative differences [10,23]. Using a framework of randomized controlled phase 2a trials we compared the treatment efficacies of 5 – 30 mg single doses of emodepside and 400 mg of albendazole to placebo against STHs. Notably, this is the first study to assess emodepside treatment efficacy via qPCR, offering a deeper understanding of emodepside’s clinical performance through DNA-based diagnostics.

### Qualitative comparison between Kato‑Katz and qPCR methods

Our results demonstrated a high concordance (≥70%) between Kato-Katz and qPCR for detecting STH infections across species and time points. Notably, a single positive result from either Kato-Katz or qPCR was sufficient to classify an individual as infected, due to the high specificity of both methods [24,25]. However, qPCR showed higher sensitivity, being twice as likely to detect STH-positive samples compared to Kato-Katz, especially during follow-up, indicating higher sensitivity. Similar findings have been reported in previous studies, which likely result from the fact that the majority of infections at follow-up are light intensity infections, which are detectable predominantly by qPCR [10,15,23,26–28]. We observed a significant number of discordant results among participants who tested positive for hookworm, both at baseline and during follow-up. Several factors may have contributed to this. One is the lower prevalence of hookworm infections detected by the Kato-Katz method, leading to missing positive cases due to possible light-intensity infections only identified by qPCR [16]. Additionally, hookworm eggs disintegrate quickly (within 60 minutes), making them harder to detect using Kato-Katz, while the DNA can still be detected by qPCR [23]. For the discordant samples (Kato-Katz positive but qPCR negative), the discrepancy could be attributed to insufficient lysis of the robust eggs or the absence of detectable DNA in the analyzed subsample. Considering Poisson distribution and the Kato-Katz cutoff of 48 eggs per gram of stool, this corresponds to a probability of 0.75%.

### Sensitivity and specificity of qPCR versus Kato-Katz for STH diagnosis

Currently, there is no universally accepted “gold standard” for diagnosing STH infections, particularly in regions with light-intensity infections [29–31]. To address this, we used Bayesian latent class modeling to estimate diagnostic performance across both STH-infected and non-infected populations of both qPCR and Kato-Katz [22]. Notably, we restricted our diagnostic comparison to *A. lumbricoides* infection status, as its data were independent of the inclusion criteria, unlike that of *T. trichiura* and hookworm. Our findings confirmed the higher sensitivity of qPCR in detecting *A. lumbricoides* infections compared to Kato-Katz [10,24]. This is particularly valuable in areas approaching STH elimination, where qPCR’s enhanced sensitivity could play a crucial role in identifying low-level infections that may be missed by microscopy.

For *A. lumbricoides* 81.4% of the Kato-Katz and qPCR results were in positive agreement. At baseline, 21.48% of the samples were Kato-Katz negative but qPCR positive. This discrepancy is likely due to the study designs not specifically including *A. lumbricoides* positive samples among those with lower positivity rates, thereby increasing the likelihood of missing negative or light infections with the Kato-Katz method, which were detected by qPCR. Another limitation we encountered was qPCR’s ability to detect residual DNA from worms or eggs, which may have led to an overestimation of positive samples [8,28,32,33]. This is reflected in qPCR’s significantly higher sensitivity (85.0%) compared to Kato-Katz (47.7%) in our sample set including negative samples, which is in accordance with other publications [10,23].

The higher sensitivity is also the reason of the discrepancy between the high number of samples being qPCR positive and Kato-Katz negative particularly pronounced in the follow-up [10,31,34].

### qPCR Ct value as a quantitative measure of STH infection

Despite the advantages of qPCR, the correlation between microscopic egg counts and qPCR Ct values in our study was moderate at best [10]. We attribute this to the low number of positive samples and the overall light intensity of infections observed, particularly for hookworm and *A. lumbricoides* Specifically, for *A. lumbricoides*, baseline samples showed fair correlation due to the higher egg output of this species, which results in consistently low Ct values and reduces variability (S2 Table).

### Comparing treatment outcomes

The possibility for qPCR to detect light infections and remaining worm DNA also leads to lower cure rates using qPCR than those obtained via Kato-Katz, especially if cure rates observed with Kato-Katz were high. Our results indicate that emodepside demonstrated significantly higher efficacy than albendazole in treating *T. trichiura* and *A. lumbricoides*, as assessed by both diagnostic methods. However, for hookworm infections, higher cure rates were observed only at emodepside doses exceeding 10 mg, possibly due to a higher number of coinfections affecting emodepside’s performance [35]. Additionally, hookworms reside within the intestinal lumen, whereas *T. trichiura* partially inhabits tissues, which could lead to a longer persistence in the intestine, leading to potentially higher false positive rates [10].

When using qPCR as the standard diagnostic tool, emodepside exhibited variable efficacy across doses, with some falling below the benchmark target product profiles established for anthelmintic drug candidates [36]. These findings suggest the need for further investigations with larger sample sizes to fully assess emodepside’s efficacy using qPCR. Regardless of the diagnostic method, emodepside consistently shows superiority in terms of cure rates compared to albendazole in treating *T. trichiura*.

### Limitations

One limitation of this study is the selection of participants, which included only those who tested positive for *T. trichiura* or hookworm at baseline, likely leading to higher concordance rates, particularly observed at baseline. Lastly, the small sample size of the follow-up data contributed to increased variability in the results, for determining efficacy of emodepside.

## Conclusion

In summary, this study underscores the importance of qPCR as a diagnostic tool for STHs, given its superior sensitivity. This tool is particularly effective in test and treat approaches for the early detection and containment of STH infections. Furthermore, standardizing both procedures will be crucial for generating more comparable and reliable results, ultimately ensuring the best possible diagnostics for patients [37]. The efficacy of emodepside is clearly demonstrated, surpassing albendazole in treating *T. trichiura*. Higher doses also proved more effective for hookworm infections, and overall excellent cure rates against *A. lumbricoides*. These findings reinforce emodepside’s potential as a game changer in the field of anthelminthics.

## Supporting information

S1 Table Properties of the primer and probes used. (DOCX)

S2 Table Mean Ct values and standard deviation obtained for qPCR analysis. (DOCX)

S1 Data Full dataset. (XLSX)

S1 STARD Checklist Checklist following STARD guidelines for reporting diagnostic accuracy studies. (DOCX)
