# Supplementary material for: Performance of real-time polymerase chain reaction and Kato-Katz for diagnosing soil-transmitted helminth infections and evaluating treatment efficacy of emodepside in randomized controlled trials
Source: PLoS Negl Trop Dis. 2025 Feb 18;19(2):e0012872. doi: 10.1371/journal.pntd.0012872 (PMC11835329; doi:10.1371/journal.pntd.0012872)
Supplement: S1 Table — (DOCX) [file pntd.0012872.s001.docx]

| **Organism** | ***T. trichura*** | ***A. lumbricoides*** | ***N. americanus*** |
| --- | --- | --- | --- |
| Target | 18S ribosomal RNA | Internal transcribed spacer 1 | Internal transcribed spacer 2 |
| Forward primer | TTGAAACGACTTGCTCATCAACTT | GTAATAGCAGTCGGCGGTTTCTT | CTGTTTGTCGAACGGTACTTGC |
| Reverse primer | CTGATTCTCCGTTAACCGTTGTC | GCCCAACATGCCACCTATTC | GTGAATAACAGCGTGCACATGTTG |
| c(primer) [uM] | 0.3 | 0.3 | 0.3 |
| Probe sequence | CGATGGTACGCTACGTGCTTACCATGG | TTGGCGGACAATTGCATGCGAT | ATTCCCGTTTAAGTGAAGA |
| c(probe) [uM] | 0.1 | 0.1 | 0.1 |
| Probe | FAM | HEX | CY5 |
| Quencher | BHQ1 | BHQ1 | BHQ2 |
| Annealing temp [°C] | 58 | 58 | 58 |
| RFU | 1500 | 800 | 100 |
| Calibration (log10(GCN)) | (CT-43.05)/-3.49 | (CT-45.76)/-3.45 | (CT-45.70)/-3.60 |
| R^2^-values | 0.9906 | 0.9913 | 0.9962 |
| Efficiency | 93.6 | 94.87 | 89.44 |
| LLOQ (GCN/µL) | 2 | 20 | 20 |

* Depicted is the target of each primer and probe, with their working concentrations and corresponding annealing temperature. Furthermore, the fluorophore and the quencher of the probes are named and the Relative Fluorescence Unit (RFU), that needs to be reached in the CFX Maestro to count as positive sample is displayed. Furthermore there the individual calibration equations and their corresponding R^2^-values. From the calibration curve, there was also determined the doubling efficacy [%], the dynamic range and LLOQ were samples can be quantified.
