# Supplementary material for: Performance of real-time polymerase chain reaction and Kato-Katz for diagnosing soil-transmitted helminth infections and evaluating treatment efficacy of emodepside in randomized controlled trials
Source: PLoS Negl Trop Dis. 2025 Feb 18;19(2):e0012872. doi: 10.1371/journal.pntd.0012872 (PMC11835329; doi:10.1371/journal.pntd.0012872)
Supplement: S2 Table — (DOCX) [file pntd.0012872.s002.docx]

| **Organism** | **Time point** | **Ct value** | **Standard deviation** ± |
| --- | --- | --- | --- |
| *T. trichiura* | Baseline | 32.84 | 7.94 |
|  | Follow-up | 19.40 | 14.34 |
| Hookworm | Baseline | 18.81 | 15.01 |
|  | Follow-up | 13.15 | 13.76 |
| *A. lumbricoides* | Baseline | 17.52 | 15.20 |
|  | Follow-up | 4.47 | 10.29 |
